# Recognition and unfolding of human telomeric G-quadruplex by short peptide binding identified from the HRDC domain of BLM helicase†

**DOI:** 10.1039/d2ra03646k

**Published:** 2022-08-08

**Authors:** Taniya Sharma, Nikita Kundu, Sarvpreet Kaur, Amlan Chakraborty, Aman Kumar Mahto, Rikeshwer Prasad Dewangan, Jadala Shankaraswamy, Sarika Saxena

**Affiliations:** Structural Biology Lab, Amity Institute of Biotechnology, Amity University Uttar Pradesh Sector-125, Expressway Highway Noida 201313 India ssaxena1@amity.edu sarikaigib@yahoo.co.in +0120-4735600; Division of Immunology, Immunity to Infection and Respiratory Medicine (DIIRM), School of Biological Sciences, University of Manchester Manchester England; Department of Pharmaceutical Chemistry, School of Pharmaceutical Education and Research, Jamia Hamdard New Delhi India; Department of Fruit Science, College of Horticulture, Sri Konda Laxman Telangana State Horticultural University Mojerla 509382 Telangana India

## Abstract

Research in recent decades has revealed that the guanine (G)-quadruplex secondary structure in DNA modulates a variety of cellular events that are mostly related to serious diseases. Systems capable of regulating DNA G-quadruplex structures would therefore be useful for the modulation of various cellular events to produce biological effects. A high specificity for recognition of telomeric G-quadruplex has been observed for BLM helicase. We identified peptides from the HRDC domain of BLM using a molecular docking approach with various available solutions and crystal structures of human telomeres and recently created a peptide library. Herein, we tested one peptide (BLM HRDC peptide) from the library and examined its interaction with human telomeric variant-1 (HTPu-var-1) to understand the basis of G4-protein interactions. Our circular dichroism (CD) data showed that HTPu-var-1 folded into an anti-parallel G-quadruplex, and the CD intensity significantly decreased upon increasing the peptide concentration. There was a significant decrease in hypochromicity due to the formation of G-quadruplex-peptide complex at 295 nm, which indicated the unfolding of structure due to the decrease in stacking interactions. The fluorescence data showed quenching upon titrating the peptide with HTPu-var-1-G4. Electrophoretic mobility shift assay confirmed the unfolding of the G4 structure. Cell viability was significantly reduced in the presence of the BLM peptide, with IC_50_ values of 10.71 μM and 11.83 μM after 72 and 96 hours, respectively. These results confirmed that the selected peptide has the ability to bind to human telomeric G-quadruplex and unfold it. This is the first report in which a peptide was identified from the HRDC domain of the BLM G4-binding protein for the exploration of the G4-binding motif, which suggests a novel strategy to target G4 using natural key peptide segments.

## Abstract 2

Schematic representation of (HTPu–var-1-G4) located at the 3′ end, formation of G-quadruplex, model of the G-quadruplex structure, base stacking between G-quadruplex planes, G-quadruplex structure-peptide complex and twisting of G-quadruplex planes upon peptide binding.

## Graphical abstract

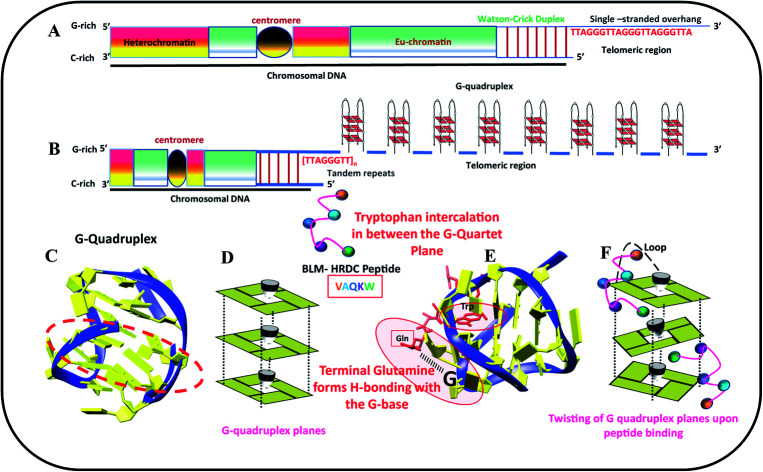

## Introduction

G-Clusters are unevenly distributed throughout the genome and can adopt various conformations with subtle differences such as parallel or anti-parallel strand orientation, or differences in the G run length or the loops of non-Gs between G runs.^1^ Recent advances in the characterization of several G-quadruplex (G4) helicases led to the view that distinct G4 structures in cells can be recognized and unwound by numerous human helicases to regulate gene functions.^2,3^

To date, the biological functions of Werner syndrome protein (WRN) and Bloom syndrome protein (BLM) have been published in many reviews, including the latest ones describing the diverse genome maintenance mechanisms of the RecQ family.^4–6^ RecQ helicases, a subfamily of DNA unwinding enzymes that belongs to the SF2 helicase superfamily, are enzymes that use ATP-driven motor force, which plays essential roles at multiple steps in DNA recombination, replication, and repair. BLM encodes one of five members of the RecQ helicase family in humans. BLM is known to resolve regions throughout the genome that are difficult to replicate, including telomeres that form a highly polymorphic G-quadruplex structure.^7^ Hence, mutations in the BLM protein are associated with rare genetic diseases such as Bloom syndrome, which is associated with high genetic instability and a high frequency of predisposition to cancer. This illustrates the primary importance of BLM in preventing tumorigenesis. In addition, Bloom syndrome patients display severe growth retardation with short stature, immunodeficiency, photosensitive skin changes, and a predisposition to a wide spectrum of cancers.^8^

Indeed, cells derived from afflicted patients show pronounced genomic instabilities such as sister chromatid exchange and telomere shortening. Telomeres are structures at the ends of chromosomes that cap the ends of chromosomal DNA from degradation, fusion, or recombination.^9,10^ Human telomeres form G-quadruplex structures using the tandem repeats of the hexanucleotide d(TTAGGG)_*n*_ that are 5–25 kb in length and terminate in a single-stranded 3′-overhang.^11,12^ These structures are stabilized with a central metal ion and guanine bases joined by Hoogsteen-type hydrogen bonds, stacked G-tetrad layers, and by hydration.^13–16^ Hence, understanding the human telomeric G-quadruplex structure under physiologically relevant conditions in K^+^ solution has been the subject of intense investigation.

It was proposed that the BLM engages in the genome-wide activity of resolving regions that are difficult to replicate, including telomeres.^17^ Hence, we studied the BLM protein, which is composed of 1417 amino acids (a.a.) with ATPase, RQC, and helicase-and-RNase-D C-terminal (HRDC) domains. It has been reported that the BLM HRDC domain preferentially binds to ssDNA with low affinity and also with Holliday junctions.^18–21^ Thus, we created a peptide library using various human telomeric structures with the BLM HRDC domain. Based on the folding topology, we segregated different crystal structures of human telomeric sequences into parallel, antiparallel, and mixed G4s. Next, we performed molecular docking with the HRDC domain of BLM to identify the small peptides binding to polymorphic G4 structures formed by human telomeres (unpublished data, filed patent).

The calculated binding energies for the BLM-2RRD domain (parallel, antiparallel, and mixed) for each G4 structure indicates that these peptides may be used as lead molecules to target polymorphic telomeric G4. Hence, these may be used as suitable natural drug molecules for targeting diseases related to telomere dysfunctions and mutations in BLM protein.

Most studies thus far involve potassium or sodium cations because of their prevalence in human cells, but a number of other monovalent and divalent cations promote quadruplex formation. However, among alkali metals, only potassium, sodium, and rubidium are truly effective at stabilizing G4s. In specific coordination to the tetrads, the cations are involved in electrostatic and donor–acceptor orbital interactions with lone pairs of guanine O6, yielding tight M+−O coordination bonds.^22^ Stabilization is also provided by electronic repulsion of these O6 lone pairs.^23^ Sodium and potassium bind G-quadruplexes with different affinities, and their binding mode is also different. It is often stated that Na^+^ is sufficiently small (0.95 Å) to fit in the plane of a quartet, while any cation larger than that, such as K^+^ (1.33 Å), is coordinated between two planes. Proteins are also present in the same ionic environment.

In the present study, we performed interaction studies between the selected peptide identified from the HRDC domain of BLM and human telomere G4 under physiological ionic conditions such as 100 mM Na^+^ or 100 mM K^+^. We selected and tested one peptide from the library and observed its interaction with human telomere variant-1-G4 (HTPu-var-1-G4) using biophysical and biochemical studies. Our circular dichroism (CD) data suggested that HTPu-var-1-G4 folded into anti-parallel G-quadruplexes, and there was a significant decrease in CD intensity upon increasing the peptide concentration under dilute conditions and cell-mimicked conditions of molecular crowding.

Our ultraviolet (UV) thermal melting results showed a significant decrease in hypochromicity upon increasing the peptide concentration, which confirmed the binding of the peptide with HTPu-var-1-G4. We observed significant quenching upon titrating the peptide with an increasing concentration of DNA with *K*_b_ values of 0.46 ± 0.11 μM, 0.71 ± 0.42 μM, and 0.51 ± 0.24 μM in the presence of 100 mM Na^+^, 100 mM K^+^, and a combination of 100 mM K^+^ and 40 wt% PEG 200, respectively. This BLM peptide showed preferential cytotoxicity when MDA-MB 241 breast cancer cells were tested, with IC_50_ values of 10.71 μM and 11.83 μM after 72 and 96 hours, respectively.

## Materials and methods

### 1. DNA and peptide

Polyacrylamide gel electrophoresis (PAGE)-purified grade DNA oligonucleotides were purchased from Helix Biosciences. The high-performance liquid chromatography (HPLC)-purified peptide VAQKW was synthesized, and its electrospray ionization-mass spectrometry (ESI-MS) spectra and HPLC chromatogram are given in Fig. S1 and S2,† respectively. The concentration of the peptide was determined by measuring the absorbance of Trp at the C-terminal at 280 nm and 25 °C. The concentrations of single-strand DNA oligonucleotides were determined by measuring the absorbance at 260 nm at a high temperature using a Shimadzu 2600 spectrophotometer (Shimadzu, Tokyo, Japan) connected to a thermoprogrammer. Single-strand extinction coefficients were calculated from mononucleotide and dinucleotide data using the nearest neighbour approximation.^24,25^

### 2. Circular dichroism spectroscopy

CD spectra were obtained with a JASCO-715 spectropolarimeter using a quartz cuvette with a 1 cm path length. All the spectra were recorded in the wavelength range of 200–350 nm at a scanning rate of 100 nm min^−1^. Before measurement, the samples were heated to 95 °C in a water bath, slowly cooled to room temperature, and then incubated at 4 °C overnight to prevent the formation of any structures under non-equilibrium conditions. Average scans of the DNA samples were subtracted from the buffer scan, and the data were normalized as a function of DNA strand concentration and path length of the cuvette.

### 3. Thermal melting analysis

The UV absorbance of different samples was recorded with a Shimadzu 2600 spectrophotometer (Shimadzu, Tokyo, Japan) equipped with a temperature controller. Melting curves of DNA structures were obtained by measuring the UV absorbance at 260 nm or 295 nm in 30 mM sodium cacodylate buffer pH (7.0) containing 0.5 mM EDTA, 100 mM NaCl, 100 mM KCl, with and without 40 wt% PEG 200 in the presence or absence of BLM HRDC peptide at different DNA : peptide ratios. The *T*_m_ values for 4 μM DNA structures were obtained from the UV melting curves as previously described.^24,25^ The heating rate was at 0.5 °C min^−1^. Before measurement, the samples were heated to 95 °C in a water bath, slowly cooled to room temperature, and then incubated at 4 °C overnight to prevent the formation of any structures under non-equilibrium conditions. Experiments were repeated in triplicate to reproduce the data.

## Thermal difference spectra

G-Quadruplex oligonucleotides were dissolved in sodium cacodylate buffer, pH 7.0, containing 100 mM KCl and 0.5 mM EDTA. The G-quadruplex samples were denatured at 95 °C for 2 min, and then were gradually cooled to room temperature overnight prior to data collection. The TDS measurements were performed using a Shimadzu 2600 spectrophotometer (Shimadzu, Tokyo, Japan) equipped with a temperature controller. The absorbance spectra were collected in triplicate at 20 °C and 95 °C in the 200–400 nm wavelength range. Thermal difference spectra were obtained by subtraction of the low-temperature from the high-temperature absorbance spectrum, and Origin 22 software was used for the spectral analysis. The differential spectra were normalized by dividing the data by their maximum values.

### 4. Native gel electrophoresis

For the native gel experiment, 15% (w/v) polyacrylamide gel was used. For the PAGE experiment, samples were prepared in 30 mM sodium cacodylate buffer (pH 7.4) containing 0.5 mM EDTA, and 100 mM NaCl or 100 mM KCl. The samples were heated to 95 °C in a water bath, slowly cooled to room temperature, and incubated at 4 °C overnight. The TBE (pH 7.4) running buffer contained the same concentration of salt and EDTA as that in the gel and also that contained in the oligonucleotide sample. The experiment was performed at 4 °C at constant voltage (50 V). A 1 : 1 mixture of glycerol and orange G dye was used for tracking the movement of DNA oligonucleotides in the gel. Finally, the gel was stained with silver using a standard protocol and imaged using the Gel-Doc system (Bio-Rad, Gurgaon, Haryana, India).

### 5. Fluorescence measurements

Fluorescence experiments were performed by utilizing a JASCO FP 8300 spectrofluorometer (JASCO, Tokyo, Japan). Experiments were carried out at 25 °C in a 1 cm path-length quartz cuvette. We used 4 μM peptide in 30 mM buffer, pH 7.0, containing 0.5 mM EDTA, 100 mM NaCl, and 100 mM KCl with and without 40 wt% PEG 200 titrated with equimolar concentrations of HTPu-var-1-G4. The temperature of the cell holder was regulated by the JASCO ETC-273T temperature controller. Samples were prepared using the same procedure. The excitation and emission slit width were 5 nm each. The samples were excited at 275 nm, and the emission was recorded in a range of 300 nm to 500 nm. The experiment was repeated in triplicate to reproduce the data. The fluorescence intensity of the BLM HRDC peptide was plotted at 357.5 nm, 356 nm, and 352.5 nm against DNA concentration in the presence of Na^+^ alone, K^+^ alone, and K^+^ alone with 40 wt% PEG 200, and fitted using the following equation. After normalization, the following equation was obtained:Y = [peptide]^*n*^/{*K*_d_^*n*^ + [peptide]^*n*^}

### 6. Electrophoretic mobility shift assay

We prepared human telomere G-quadruplex (HTPu var-1) in 100 mM sodium cacodylate buffer, pH 7.0, with 100 mM K^+^ and 0.5 mM EDTA (Lane 2). We prepared i-motif structures using a human telomere c-rich strand (HTPy-var-1 5′-ATCCCAATCCCAATCCCAATCCC-3′) as a control in 100 mM sodium cacodylate buffer, pH 7.0 (Lane 3). Lane 1, 10-bp ladder; Lane 2, human telomere variant 1-G4: C4 (duplex); Lane 3, human telomere variant 1-C4 (i-motif (pH 7.0); Lane 4, human telomere variant-1-G4 (G-quadruplex); Lane 5, human telomere variant-1-G4 (G-quadruplex) : peptide (1 : 120) complex; Lane 6 (G-quadruplex) : peptide (1 : 120) complex incubated with a doubled concentration of HTPy; Lane 7 (G-quadruplex) : peptide (1 : 160) complex incubated with a doubled concentration of HTPy; Lane 8 (G-quadruplex) : peptide (1 : 160) complex incubated with a doubled concentration of HTPy. It is important to note that first we prepared the G4 structure, and then peptide was added the next day, followed by incubation for many hours. HTPy-var-1 was added the next day in these samples in double concentration to confirm the fact that if peptide destabilized the G-quadruplex, then the addition of HTPy-var-1 should allow the formation of duplex.

## Results and discussion

### 7. Identification of peptides from the HRDC domain of BLM

**Fig. 1:**
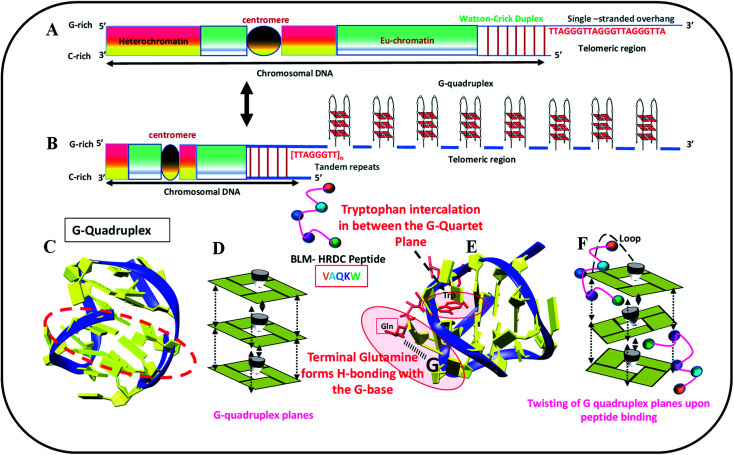
Schematic representation of human telomere (HTPu–var-1-G4 sequence) located at the (A) end, (B) formation of unimolecular G-quadruplex at ends, (C) model of G-quadruplex structure, (D) base stacking between G-quadruplex planes, (E) G-quadruplex structure-peptide complex, and (F) twisting of G-quadruplex planes upon peptide binding. Binding of peptide through loop in G-quadruplex followed by the interaction of tryptophan.

We identified biologically active peptide sequences from the HDRC domain of BLM by molecular docking with various telomeric crystals and NMR structures, and created a peptide library. In this study, we tested one peptide selected from the library to confirm its activity with the 23-mer human telomeric variant sequence (5′-TAGGGTTAGGGTTAGGGTTAGGG-3′, named as HTPu-variant-1-G4). The length of this peptide is a 5-mer, and the sequence is N-VAQKW-C. We propose that this peptide may recognize the G-quadruplex through the loop region of the G-quadruplex, followed by the intercalation of tryptophan between the G-quartet plane. This mode of binding may allow the G-quartet planes to twist over one another, which may decrease the stacking interactions and hence allow the HTPu–var-1-G4 to unfold. Fig. 1 schematically shows the location of human telomeres (HTPu–var-1-G4 sequence) at the ends, the formation of the G-quadruplex structure at the ends, the stacking of G-quadruplex planes, the G-quadruplex-peptide complex, and the twisting of quadruplex-peptide complex planes upon peptide binding.

Structural changes in the human telomeric variant sequence in the presence of Na^+^, K^+^ and K^+^ with 40 wt% PEG 200 with and without peptide under dilute conditions and cell-mimicked conditions of molecular crowding.

**Fig. 2:**
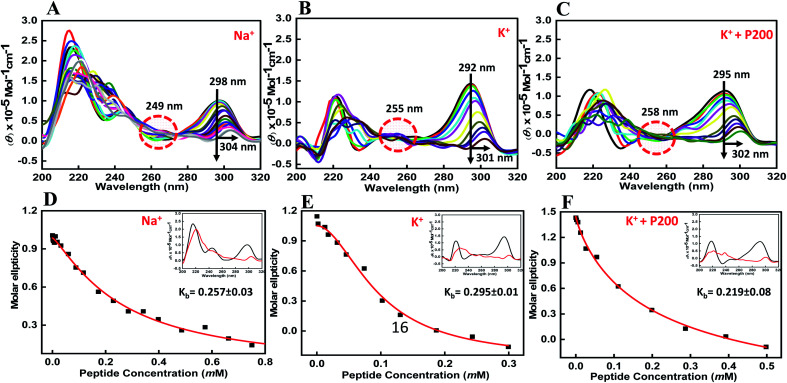
CD spectra of 6 μM human telomere variant 1-G4 and their respective binding constant values (*K*_b_) in buffer containing (A and D) 0.5 mM EDTA and 100 mM NaCl, (B and C) 100 mM KCl, and (C and F) 100 mM KCl with 40 wt% PEG 200, without any additive (black line) and titrated with an increasing concentration of BLM HRDC peptide.

CD spectroscopy is one of the simplest methods that can be used to characterize the G-quadruplex topology because G-quadruplex structures with different polarities exhibit different CD spectral characteristics.^26,27^ The ability of the BLM HRDC peptide to bind HTPu–var-1-G4 was assessed by CD spectroscopy in the presence of either sodium or potassium ions. To directly compare the DNA structures with and without peptide, first we recorded the CD structure of HTPu–var-1-G4 under dilute conditions. The structure of the HTPu–var-1-G4 strand was recorded in the presence of 30 mM sodium cacodylate buffer, pH 7.0, containing 100 mM Na^+^ (Fig. 2a) or 100 mM K^+^ (Fig. 2b) and 100 mM K^+^ with 40 wt% PEG 200 (Fig. 2c) in the presence and absence of peptide and studied by CD and non-denaturing PAGE (Fig. S3†).

We found that the CD spectrum of HTPu–var-1-G4 in 100 mM Na^+^ was characterized by a positive peak at 298 nm and negative peak at 238 nm, which is typically observed for antiparallel G-quadruplexes in the presence of Na^+^^28,29^ (Fig. 2a). Next, it was titrated with an increasing concentration of the BLM peptide to determine the bound conformation of the peptide with a G-quadruplex structure. We observed a decrease in CD intensity with a 6 nm redshift along with an isodichroic point at 249 nm, which indicated that the peptide was able to bind to the HTPu–var-1-G4 structure. Similarly, the CD spectrum of HTPu–var-1-G4 in 100 mM K^+^ was characterized by a positive peak at 292 nm and prominent negative peak at 238 nm, indicating the formation of antiparallel G-quadruplexes.^28,29^

On titrating with an increasing concentration of peptide, there was a significant decrease in CD intensity, and the positive peak shifted to 301 nm along with the isodichroic points at 229 nm and 254 nm, which suggested that a conformational conversion of G-quadruplex into G-quadruplex-peptide complex occurred. We also confirmed the changes in its structure in the presence of K^+^ upon peptide binding. The CD spectrum was characterized by a positive peak at 295 nm and a negative peak at 238 nm, which represents the antiparallel G-quadruplex in the presence of 100 mM K^+^ and 40 wt% PEG 200 (Fig. 2c). There was a continuous decrease in CD intensity, and the positive peak shifted to 302 nm along with the isodichroic point at 258 nm.

These CD results confirmed that HTPu–var-1-G4 was able to bind with BLM in the absence of peptide and significantly unfolded the G-quadruplex structure. The apparent binding constant (*K*_b_) in 100 mM Na^+^ (Fig. 2d), 100 mM K^+^ (Fig. 2e), and 100 mM K^+^ with 40 wt% PEG 200^+^ (Fig. 2f) was 0.26 ± 0.03 mM, 0.29 ± 0.01 mM, and 0.22 ± 0.08 mM, respectively. The possibility of G-quadruplex destabilization will be further explored by UV thermal melting and native gel electrophoresis data in the following sections.

### 8. UV melting studies of human telomeric G-quadruplex with and without peptide

**Fig. 3:**
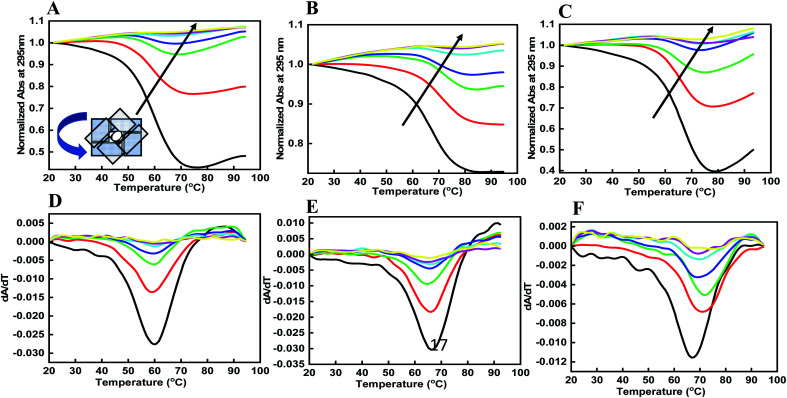
Normalized UV melting curves of 4 μM human telomere var-1-G4 with their derivatives without any additive (black line), human telomere var-1-G4: BLM HRDC peptide ratio (1 : 20), (1 : 10), and (1 : 20) (redline), (1 : 40), (1 : 20), (1 : 40) (green line), (1 : 80), (1 : 40), (1 : 80) (blue line), (1 : 160), (1 : 80), (1 : 160) (turquoise line), (1 : 230), (1 : 120) (1 : 230) (red line), and (1 : 310), (1 : 160), (1 : 310) (yellow line) in buffer containing (A and D) 0.5 mM EDTA and 100 mM NaCl, (B and C) 100 mM KCl, and (C and F) 100 mM KCl with 40 wt% PEG 200.

DNA quadruplexes show little change in absorbance at their UV maximum (260 nm), and greater signal at 295 nm.^29^ Hence, we explored the thermal stability of the DNA structures with and without peptide at 295 nm. Fig. 3 shows a normalized UV melting profile and derivatives of 4 μM HTPu–var-1-G4 in buffer containing 100 mM NaCl, (Fig. 3a, d), KCl (Fig. 3b, e), and KCl + 40 wt% PEG 200 (Fig. 3c, f) in the absence and presence of BLM HRDC peptide. The ratios of HTPu–var-1-G4 and BLM peptide were 1 : 0, 1 : 10, 1 : 20, 1 : 40, 1 : 80, 1 : 120, and 1 : 160 (Fig. 3a).

The melting temperature (*T*_m_) was evaluated by a previously described curve-fitting procedure.^24,25^ Melting curves with a single transition were obtained under all conditions. The *T*_m_ values were recorded as 60.6 °C for the control, which decreased to 59.7 °C in Na^+^ (Fig. 3a); 64.3 °C for the control, which decreased to 63.3 °C in K^+^ (Fig. 3b); and 67.6 °C for the control, which remained at 67.6 °C in K^+^ with 40 wt% PEG 200 (Fig. 3c and Table 1). The change in Δ*H*° was −18.84 kcal mol^−1^ to 18.12 kcal mol^−1^, −19.83 kcal mol^−1^ to −20.09 kcal mol^−1^, −20.31 kcal mol^−1^; the change in *T*Δ*S*° was −242.76 kcal mol^−1^ to −238.58 kcal mol^−1^, −244.93 kcal mol^−1^ to −245.32 kcal mol^−1^, and −253.16 kcal mol^−1^ to −253.09 kcal mol^−1^; and the change in Δ*G*° was −6.92 kcal mol^−1^ to −6.21 kcal mol^−1^, −8.93 kcal mol^−1^ to −6.91 kcal mol^−1^, and −11.32 kcal mol^−1^ to −11.03 kcal mol^−1^ at a DNA : peptide ratio of (1 : 0) to (1 : 230), (1 : 0) to (1 : 120), and (1 : 0 to 1 : 160) in the presence of Na^+^, K^+^, and K^+^ + 40 wt% PEG 200, respectively.

**Table 1:** UV melting curve-derived thermodynamic parameters at 25 °C for human telomere var-1-G4 with and without BLM HRDC peptide at pH 7.4

Abbreviation	*T*_m_/°C	Δ*H*°/kcal mol^−1^	*T*Δ*S*°/kcal mol^−1^	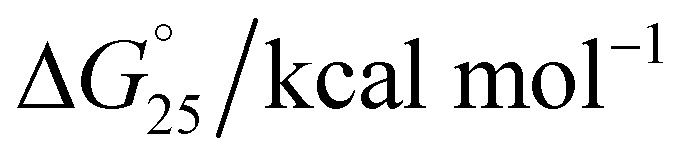
**Na** ^**+**^				
HTPu-Var1 : Pep (1 : 0)	60.6	−18.84 ± 0.20	−242.76 ± 0.34	−6.92 ± 0.12
HTPu-Var1 : Pep (1 : 20)	59.7	−17.64 ± 0.23	−238.652 ± 0.12	−5.32 ± 0.56
HTPu-Var1 : Pep (1 : 40)	59.7	−17.82 ± 0.31	−238.78 ± 0.32	−5.278 ± 0.32
HTPu-Var1 : Pep (1 : 80)	58.8	−19.12 ± 0.29	−237.813 ± 0.24	−5.72 ± 0.36
HTPu-Var1 : Pep (1 : 160)	59.7	−18.78 ± 0.21	−241.69 ± 0.12	−6.13 ± 0.27
HTPu-Var1 : Pep (1 : 230)	59.7	−18.12 ± 0.42	−238.58 ± 0.23	−6.21 ± 0.43

**K** ^**+**^				
HTPu-Var1 : Pep (1 : 0)	64.3	−19.83 ± 0.14	−244.93 ± 0.64	−8.93 ± 0.12
HTPu-Var1 : Pep (1 : 10)	63.3	−18.89 ± 0.43	−243.039 ± 0.42	−7.83 ± 0.53
HTPu-Var1 : Pep (1 : 20)	63.3	−19.001 ± 0.18	−243.78 ± 0.32	−7.47 ± 0.19
HTPu-Var1 : Pep (1 : 40)	63.3	−19.92 ± 0.26	−243.09 ± 0.74	−7.92 ± 0.42
HTPu-Var1 : Pep (1 : 80)	66.1	−20.85 ± 0.25	−253.69 ± 0.38	−9.432 ± 0.29
HTPu-Var1 : Pep (1 : 120)	64.3	−18.45 ± 0.51	−245.32 ± 0.53	−6.918 ± 0.33

**K** ^**+**^ **+P200**				
HTPu-Var1 : Pep (1 : 0)	67.6	−20.09 ± 0.43	−253.162 ± 0.34	−11.32 ± 0.77
HTPu-Var1 : Pep (1 : 20)	69.5	−21.42 ± 0.21	−273.28 ± 0.45	−12.72 ± 0.63
HTPu-Var1 : Pep (1 : 40)	69.5	−21.392 ± 0.65	−273.92 ± 0.26	−12.28 ± 0.34
HTPu-Var1 : Pep (1 : 80)	67.6	−20.28 ± 0.23	−252.96 ± 0.19	−10.98 ± 0.45
HTPu-Var1 : Pep (1 : 160)	67.6	−20.31 ± 0.17	−253.09 ± 0.48	−11.03 ± 0.54

Interestingly, there was a drastic decrease in the extent of hypochromicity under all three ionic conditions, which clearly indicated that unfolding of the human telomeric G-quadruplex had occurred under dilute conditions and mimicked conditions of molecular crowding. It is generally accepted that the stacking between G-quartets provides stability to a G-quadruplex structure, whereas the size and the orientation of the loops determine the type and flexibility of the structure.^30^ Individual G-quadruplex units have the capacity to stack on one another.^31^ Therefore, we propose that peptides binding to the HTPu–var-1-G4 unfolded the structure due to peptide binding on the loop region of the G4 structure. This might affect the stacking interactions between the G-quartet plane due to the intercalation of tryptophan located in the terminus that allowed the planes to twist over one another and hence unfold the structure. However, this has to be investigated by detailed structural analysis using NMR or X-ray crystallography.

We also calculated the thermal difference spectrum (TDS) by the mathematical subtraction of two spectra recorded at a temperature above the *T*_m_ (95 °C) and at a low temperature (20 °C) (Fig. S3†). At high temperature, the oligonucleotide is fully dissociated (the fraction folded, *θ* = 0), and at lower temperature, the oligonucleotide is fully associated. The shape of this difference spectrum was found to be a signature for quadruplexes. Mergny *et al.* reported that the TDS of the G-quadruplex structure has a specific shape with two positive signals at approximately 243 and 273 nm and a negative signal near 295 nm.^32^ We observed a similar structure in the TDS spectra for HTPu–var-1-G4, which showed the G-quadruplex formation. Interestingly, this signature changed after adding the peptide under similar solution conditions. These data confirmed the binding of peptide with the HTPu–var-1-G4 structure and its unfolding due to peptide binding.

### 9. CD melting studies of human telomeric G-quadruplex with and without peptide

We also performed CD melting experiments using HTPu–var-1-G4 with and without peptide in 100 mM KCl and with the different DNA : peptide ratios of 1 : 0, 1 : 10, 1 : 40, 1 : 80, and 1 : 100. We recorded the changes in the temperature-dependent spectra at 5 °C intervals, starting from 20 °C to 95 °C, and plotted the change in CD intensity against temperature (Fig. S4†). We did not observe any significant change in the *T*_m_ values, but the percentage of hypochromicity decreased upon increasing the peptide concentration, which was consistent with our UV melting results. These melting results confirmed the binding of peptide with G-quadruplex and its unfolding possibly due to the decrease in stacking interaction.

### 10. Changes in the molecularity of HTPu–var-1-G4 upon peptide binding using native gel electrophoresis

To reveal the molecular mechanism of the binding of HTPu–var-1-G4 and the BLM HRDC peptide, we further investigated the complex in the presence of Na^+^ or K^+^ using non-denaturing PAGE (Fig. S5†). The PAGE experiment can discriminate the molecularity of the HTPu–var-1-G4 and HTPu–var-1-G4-peptide complex. The electrophoretogram in Fig. S5† shows the structural status of HTPu–var-1-G4 in the presence and absence of the BLM HRDC peptide. A 10-bp DNA ladder was used to compare their electrophoretic mobility.

Lane 2 shows that the migration of a single band was equivalent to that of 10 base pairs, which indicated that HTPu–var-1-G4 folded into a unimolecular G4 structure. Lanes 3 to 6 show one band that is equivalent to 10 bp. We assigned this band to the G-quadruplex peptide complex. There was no change in band intensity or band position because a single purine-rich strand and unimolecular G-quadruplex will both migrate at similar positions. Hence, we designed another experiment to distinguish the unimolecular G-quadruplex and single purine-rich strand generated after peptide binding.

**Fig. 4:**
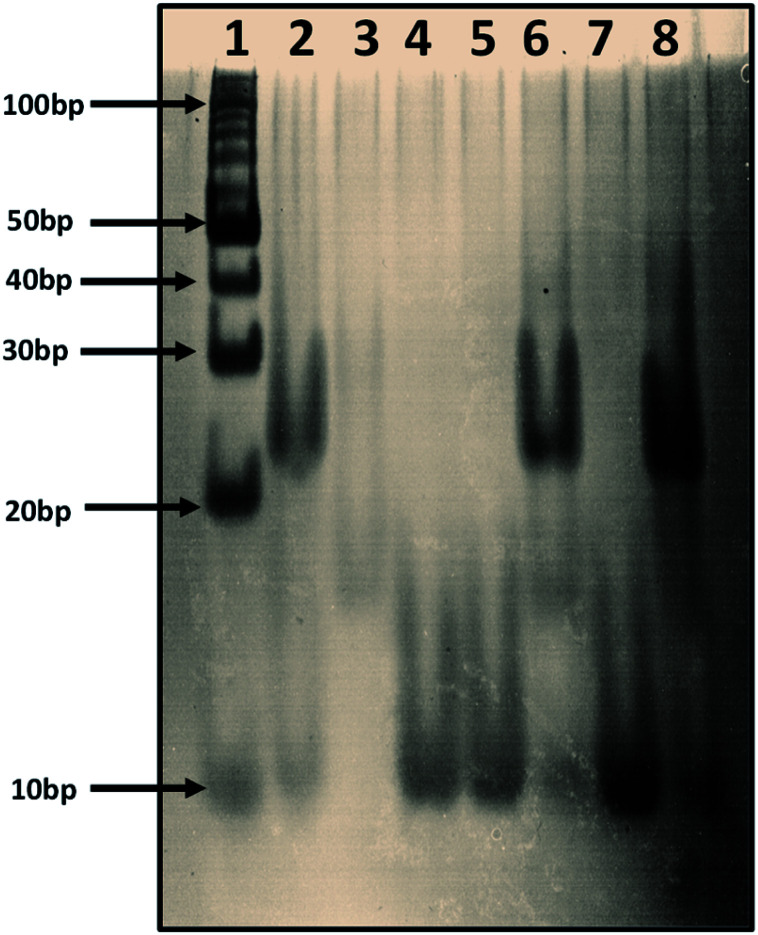
Electrophoretic mobility shift assay of 4 μM human telomere variant 1-G4 in 30 mM sodium cacodylate buffer (pH 7.0), 1 mM EDTA, 100 mM KCl. Lane 1, 10-bp ladder; Lane 2, human telomere variant 1-G4: C4 (duplex); Lane 3, human telomere variant 1-C4 (i-motif (pH 7.0); Lane 4, human telomere variant 1-G4 (G-quadruplex); Lane 5, human telomere variant 1-G4; Lane 6, (G-quadruplex) : peptide (1 : 120) complex incubated with double concentration of HTPy; Lane 7, (G-quadruplex) : peptide (1 : 160) complex incubated with double concentration of HTPy; Lane 8 (G-quadruplex) : peptide (1 : 160) complex incubated with double concentration of HTPy.

To confirm the unfolding of human telomere HTPu–var-1-G4 upon peptide binding in K^+^, we performed an electrophoretic mobility shift assay. In the electrophoretogram of Fig. 4, a 10-bp DNA ladder was used to compare their electrophoretic mobility. Lane 2 displays two bands, where the upper band migrated between 20 bp and 30 bp, which indicated the formation of DNA duplex, while the lower faint band appeared near 10 bp, which corresponded to the free single strand. Lane 3 displays single bands that migrated between 10 and 20 base pairs, which indicates the formation of dimeric i-motif structures both at pH 7.0, which was consistent with the results from our recently published paper on i-motif. Lane 4 displays a single band equivalent to 10 base pairs, which indicates that HTPu–var-1-G4 folded into a unimolecular G-quadruplex structure.

Next, we prepared four sets as controls containing high concentrations of peptide with DNA; *e.g.*, the DNA : peptide ratios in Lane 5 and Lane 7 were 1 : 120 and 1 : 160, respectively. In these sets, we prepared the G-quadruplex structure by heating the purine-rich DNA strand to 95 °C, followed by slowly cooling to room temperature and incubating at 4 °C for many hours. We initially formed a stable G-quadruplex, and then, we added peptide to all four sets and incubated for long hours to confirm that the peptide was able to bind to the G4 structure. A single band near 10 bp is clearly seen in Lanes 5 and 7.

In two additional sets after peptide incubation, we added double the concentration of the pyrimidine-rich strand (Lanes 6 and 8). Interestingly, we observed three bands, and we assigned the upper band to a duplex, the middle band to a dimeric i-motif, and the lower band to a free single strand. However, the intensity of the upper band was greater in Lane 8 as compared to Lane 6, with the main difference being the concentration of peptide. The DNA : peptide ratios of the samples in Lane 6 and 7 were 1 : 120 and 1 : 160, respectively.

These results clearly confirmed that the peptide was binding with HTPu–var-1-G4 and unfolded the structure. We propose that the free single G-rich strands remaining after G-quadruplex unfolding were due to peptide binding, and were able to bind with pyrimidine-rich strands to subsequently form the DNA duplex. These gel results clearly confirmed that the BLM HRDC peptide has the ability to unfold the human telomeric G-quadruplex, which is consistent with our CD and UV melting results.

### 11. Exploration of peptide binding to HTPu–var-1-G4 DNA by fluorescence measurements

**Fig. 5:**
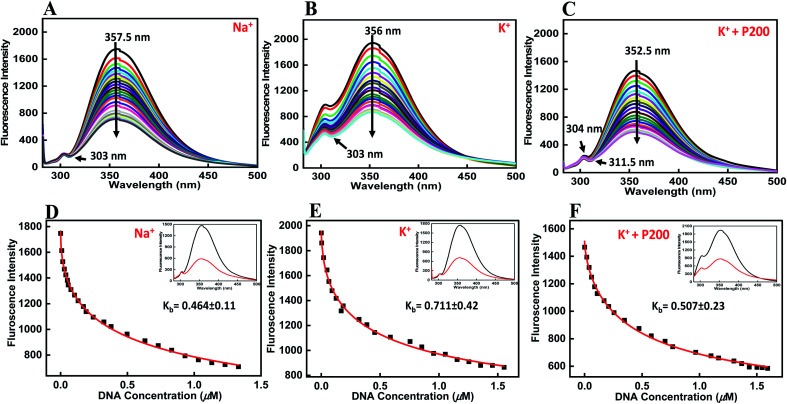
Emission spectra of BLM HRDC peptide with human variant-1-G4 and their binding constant values (*K*_b_) in buffer (pH 7.0) containing (A and D) 0.5 mM EDTA and 100 mM NaCl, (B and F) 100 mM KCl, and (C and F) 100 mM KCl with 40 wt% PEG 200 at 25 °C. BLM HRDC peptide (4 μM) was titrated with increasing concentrations of an equimolar amount of preformed human variant-1-G4.

Fluorescence titration experiments were employed to study the binding affinities of peptide with HTPu-var-1-G4. To determine the emission spectrum of a particular fluorochrome, the wavelength of maximum absorption (usually the same as the excitation maximum) is determined, and the fluorochrome is excited at that wavelength. The BLM HRDC peptide contains tryptophan, and hence, its emission spectra were examined by exciting at 275 nm. Upon excitation at 275 nm, the peptide produced an emission band due to the presence of tryptophan residues, with a maxima centred at 357.5 nm (Fig. 5a).

The HTPu-var-1-G4 was prepared in the presence of Na^+^ and was added to the peptide until very small changes in fluorescence spectra were observed. Upon addition of DNA, minor changes in the fluorescence intensity were observed due to the binding of BLM HRDC peptide with HTPu–var-1-G4, which resulted in the generation of a G-quadruplex-peptide complex. We observed that the fluorescence of the BLM HRDC peptide was quenched with a maxima peak that shifted to 354 nm upon increasing the DNA concentration and the emergence of new minima peak at 303 nm, which indicated that tryptophan intercalation within the G-quadruplex planes occurred during binding.

The binding constant *K*_b_ value was determined to be 0.46 ± 0.11 μM (Fig. 5d). Furthermore, to understand the effect of cation on the binding affinity of BLM HRDC peptide with HTPu-var-1-G4, we also performed a fluorescence titration experiment in buffer containing 100 mM K^+^ (Fig. 5b). The fluorescence maxima at 356 nm was quenched and shifted to 354 nm, and the binding constant (*K*_b_) value was determined to be 0.71 ± 0.42 μM (Fig. 5e).

Next, we recorded the fluorescence data with K^+^ and 40 wt% PEG 200 (Fig. 5c). For the fluorescence maxima at 352.5 nm with a shoulder peak at 304 nm, both peaks were quenched, and the major peak shifted to 351.5 nm with the emergence of a new minima at 311.5 nm. The binding constant (*K*_b_) value was evaluated as 0.51 ± 0.23 μM (Fig. 5f). These results further confirmed the formation of a G4-peptide complex under dilute conditions and cell-mimicked conditions of molecular crowding, with affinity of the peptide toward the G-quadruplex structure.

### 12. Inhibition of proliferation in MDA-MD-231 human breast adenocarcinoma cells

Many reports have recently suggested that small molecules identified G-quadruplexes and exhibited antiproliferative activity against human cancer cell lines, for example, AS1411, and modified thrombin-binding aptamer variants AT11, G4-STAT3, G4-TOP1, G4-SP1, G4-VEGF, G4-NCL, G4-SHP-2, and G4-TGT.^32–37^ Such G-rich molecules can bind to various proteins involved in many cellular pathways, particularly cell proliferation or apoptosis, and dysregulate their biological functions.^38^

**Fig. 6:**
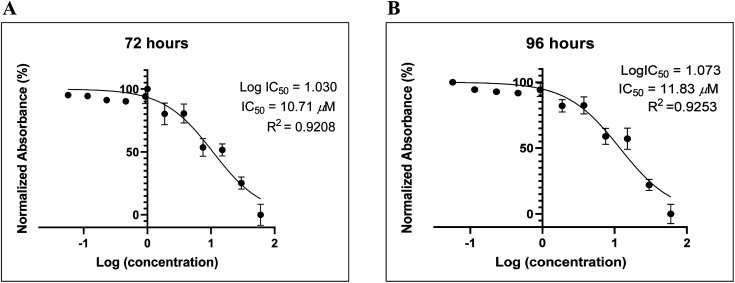
The BLM HRDC peptide demonstrated the anti-proliferative effects on MDA-MB-231 human breast adenocarcinoma cells. (A) Difference in absorbance is due to the different rate of formazan formation at two time points (24 h and 96 h) for cells incubated with BLM HRDC peptide at concentrations ranging from 40 μM to 39 nM. The data were analysed by comparing the changes in the absorbance from cells with media only (control).

Recently, we reported the anti-proliferative activity of designed peptide QW10 after binding to the c-Myc promoter G-quadruplex.^39^ Herein, to evaluate the potential of the BLM HRDC peptide for future development as a potent drug to increase the replication efficiency in patients facing genomic instability and to understand the structure–activity relationship, we examined the capacity of the peptide to inhibit the growth of human breast adenocarcinoma cells (MDA-MB-231). This technique allowed assessment of the cell viability based on the reduction of the water-soluble, yellow tetrazole salt (MTT) into insoluble dark blue formazan.^40^ The amount of reduced MTT is directly proportional to the number of living cells, and The MDA-MB-231 cell viability was significantly decreased in the presence of BLM peptide, with IC_50_ values of 10.71 μM and 11.83 μM after 72 and 96 hours, respectively (Fig. 6).

In conclusion, telomeric G4 DNA is one of the most abundant G4s in the nucleus, and the formation of telomeric DNA G4s can play an important role in telomere maintenance. Our experimental findings indicate that there is an unfolding of human telomere variant G4 caused by natural peptide extracted from the HRDC domain of the BLM protein. The results suggest that peptide bound and subsequently unfolded the G4 structure not only under dilute conditions but also under molecular crowding conditions. This unfolding might be due to a decrease in stacking interactions between G-quartet planes due to twisting of the planes upon peptide binding. Our CD and UV melting data, and electrophoretic mobility shift assay also confirmed the unfolding of the G4 structure.

We anticipate that further investigation will be required to ascertain the detailed structure of HTPu–var-1-G4 and the HRDC BLM peptide. An understanding of the detailed mode of its binding with the G-quadruplex structure will be obtained using nuclear magnetic resonance (NMR) spectroscopy or X-ray crystallography, and that work is currently in progress. This approach will identify some lead natural peptides selected from naturally occurring G-quadruplex binding proteins, which can be targeted for increasing the replication efficiency and improving telomere functioning in the future. We propose that peptides might be satisfactory candidates for targeting the G-quadruplex because they are next-generation molecules that are easy to design and synthesize, they mimic natural protein–G-quadruplex interactions, and they can easily be chemically modified for future development as a functional molecule to increase the cellular uptake for targeted delivery.

## Funding

We thank the Department of Biotechnology (DBT), Government of India for research funding for this project (SAN No. 102/IFD/SAN/864/2018-2019).

## Conflicts of interest

The authors declare no competing financial interests.

## Supplementary Material

RA-012-D2RA03646K-s001
