# Supplementary material for: Recognition and unfolding of human telomeric G-quadruplex by short peptide binding identified from the HRDC domain of BLM helicase
Source: RSC Adv. 2022 Aug 8;12(34):21760–9. doi: 10.1039/d2ra03646k (PMC9358547; doi:10.1039/d2ra03646k)
Supplement: RA-012-D2RA03646K-s001 [file RA-012-D2RA03646K-s001.pdf]

Electronic Supplementary Material (ESI) for RSC Advances.
This journal is © The Royal Society of Chemistry 2022

Recognition and Unfolding of Human Telomeric G-quadruplex by short peptide binding identified from HRDC domain of BLM Helicase

Taniya Sharma[a], Nikita Kundu[a], Sarvpreet Kaur[a], Amlan Chakraborty[b], Aman Kumar Mahto[b], Rikeshwer Prasad Dewangan[b], Jadala Shankaraswamy[d], Sarika Saxena[a*]

[a]Amity Institute of Biotechnology, Amity University Uttar Pradesh, Noida 201313, India.

[b]Division of Immunology, Immunity to Infection and Respiratory Medicine (DIIRM), School of Biological Sciences, University of Manchester.

[c]Department of Pharmaceutical Chemistry, School of Pharmaceutical Education and Research, Jamia Hamdard.

[d]Department of Fruit Science, College of Horticulture, Mojerla, Sri Konda Laxman Telangana State Horticultural University, 509382, Telangana, India.

Note: Taniya Sharma, Nikita Kundu, and Sarvpreet Kaur share equal contribution.

Supporting Information

Table of Contents **1-2**

1. **Figure S1** ESI spectra of peptide and **Figure S2** HPLC chromatogram **3**

2. **Figure S3** Thermal difference spectra of 4 μM HTPu-Var-1 G4 in buffer containing 100 mM KCl with and without Peptide (1:40) ratio. **4**

3. **Figure S4** Temperature dependent CD spectra of 4μM HTPu-Var-1 G4 in buffer containing 100 mM KCl with and without Peptide DNA: Peptide (1:0) (A), (1:10) (B), (1:40) (C), (1:80) (D), 1:100 (E), comparative CD melting profile of all DNA:Peptide ratio (F) ratio. **5**

4. **Figure S5:** Native gel electrophoresis of 6 μM HTPu-Var-1 G4 in buffer containing 100 mM KCl and DNA: Peptide ratios (1:0) Lane 2 (1:10), Lane 3 (1 : 20), Lane 4 (1 : 40), Lane 5 (1 : 80), Lane 6 (1 : 120) and (1:160) Lane 7 respectively. **6**

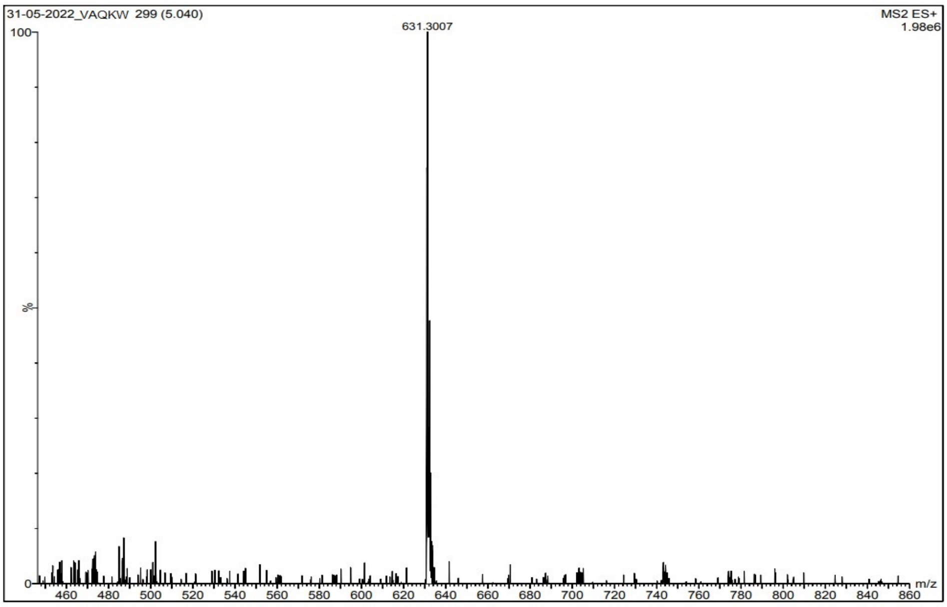

Fig. S1 ESI mass spectra of VAQKW

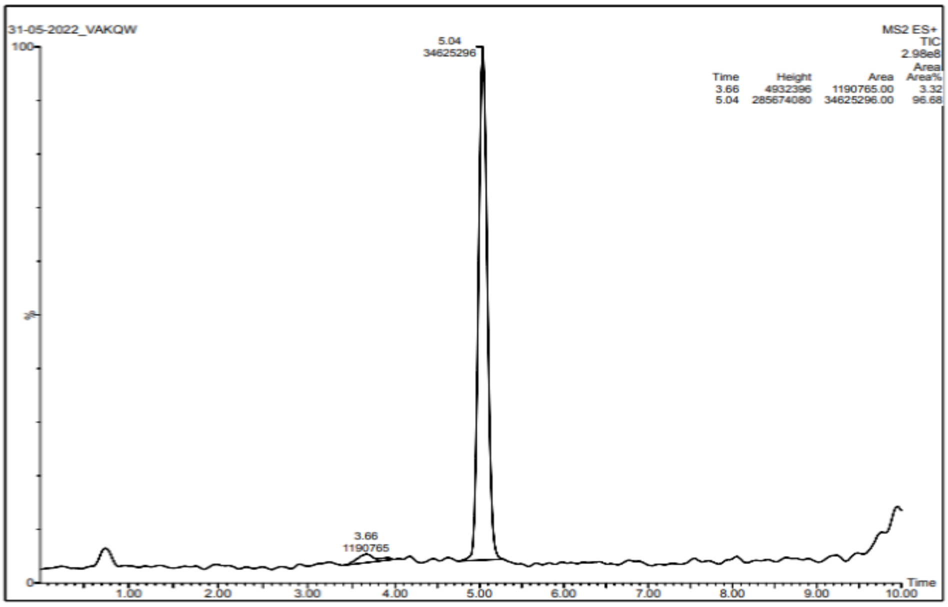

Activate Win

Fig. S2 HPLC chromatogram of VAQKW

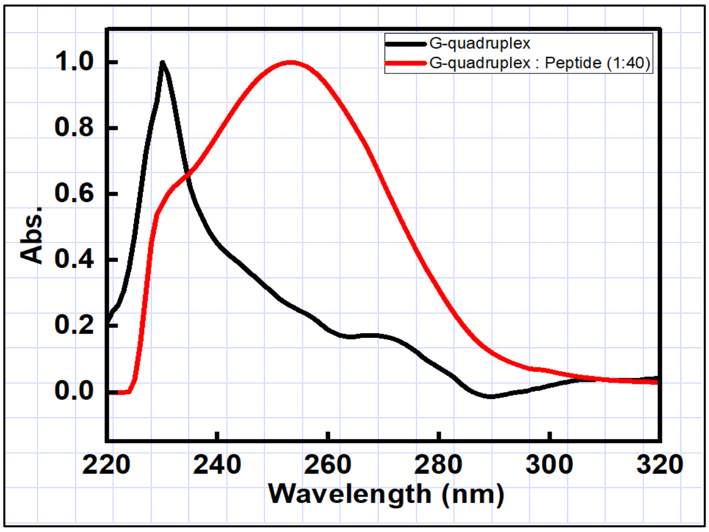

Fig. S3 Thermal difference spectra of HTPu-Var-1 G4 obtained after subtracting the lower temperature (20°C) curve from higher temperature curve (95°C) without peptide (black) and DNA:Peptide complex (1:40) red.

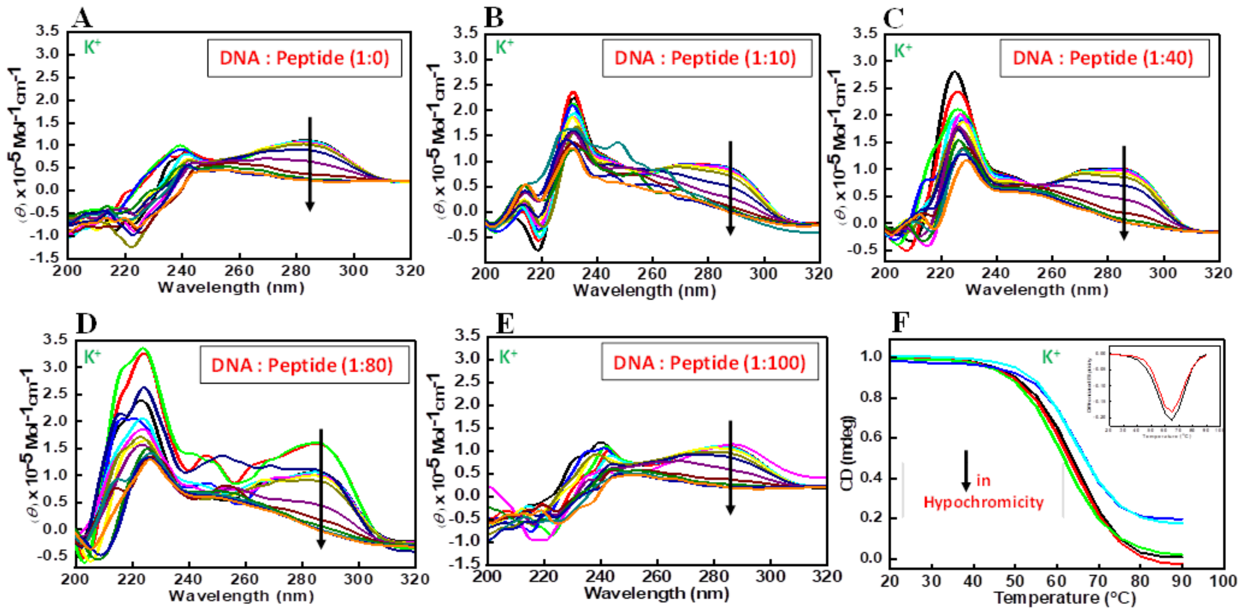

Fig. S4 Temperature dependent CD spectra of HTPu-Var-1 G DNA: Peptide (1:0) (A), (1:10) (B), (1:40) (C), (1:80) (D), (1:100) (E). Comparative CD melting profile of all DNA :Peptide ratio (F).
